# Image-based hemodynamic simulations for intracranial aneurysms: the impact of complex vasculature

**DOI:** 10.1007/s11548-023-03045-3

**Published:** 2024-01-11

**Authors:** Franziska Gaidzik, Jana Korte, Sylvia Saalfeld, Gábor Janiga, Philipp Berg

**Affiliations:** 1 Research Campus STIMULATE, Otto-von-Guericke-University Magdeburg, Magdeburg, Germany; 2 Laboratory of Fluid Dynamics and Technical Flows, Forschungscampus STIMULATE, Otto-von-Guericke-University Magdeburg, Universitätsplatz 2, 39106 Magdeburg, Germany; 3 Laboratory of Simulation and Graphics, Otto-von-Guericke-University Magdeburg, Magdeburg, Germany; 4 Department of Medical Engineering, Otto-von-Guericke-University Magdeburg, Magdeburg, Germany

**Keywords:** Circle of Willis, Computational fluid dynamics, Hemodynamics, Intracranial aneurysms, Segmentation

## Abstract

**Purpose:**

Hemodynamics play an important role in the assessment of intracranial aneurysm (IA) development and rupture risk. The purpose of this study was to examine the impact of complex vasculatures onto the intra-vessel and intra-aneurysmal blood flow.

**Methods:**

Complex segmentation of a subject-specific, 60-outlet and 3-inlet circle of Willis model captured with 7T magnetic resonance imaging was performed. This model was trimmed to a 10-outlet model version. Two patient-specific IAs were added onto both models yielding two pathological versions, and image-based blood flow simulations of the four resulting cases were carried out. To capture the differences between complex and trimmed model, time-averaged and centerline velocities were compared. The assessment of intra-saccular blood flow within the IAs involved the evaluation of wall shear stresses (WSS) at the IA wall and neck inflow rates (NIR).

**Results:**

Lower flow values are observed in the majority of the complex model. However, at specific locations (left middle cerebral artery 0.5 m/s, left posterior cerebral artery 0.25 m/s), higher flow rates were visible when compared to the trimmed counterpart. Furthermore, at the centerlines the total velocity values reveal differences up to 0.15 m/s. In the IAs, the reduction in the neck inflow rate and WSS in the complex model was observed for the first IA (IA-A δNIRmean =  − 0.07ml/s, PCA.l δWSSmean =  − 0.05 Pa). The second IA featured an increase in the neck inflow rate and WSS (IA-B δNIRmean = 0.04 ml/s, PCA.l δWSSmean = 0.07 Pa).

**Conclusion:**

Both the magnitude and shape of the flow distribution vary depending on the model’s complexity. The magnitude is primarily influenced by the global vessel model, while the shape is determined by the local structure. Furthermore, intra-aneurysmal flow strongly depends on the location in the vessel tree, emphasizing the need for complex model geometries for realistic hemodynamic assessment and rupture risk analysis.

**Supplementary Information:**

The online version contains supplementary material available at 10.1007/s11548-023-03045-3.

## Introduction

The circle of Willis (CoW) and its hemodynamics provide a representation of the global intracranial vascularization structure [1]. Knowledge about cerebral vascularization is beneficial in numerous ways for the medical community. Perosa et al. [2] have investigated the influence of hippocampal vascularization patterns on cognitive performance, but little is known about the relationship between cerebral blood flow, vascular profiles and cognition. The CoW is also a common location for the development of intracranial aneurysms (IAs) that can lead to fatal consequences when ruptured [3].

High variability in the vascular anatomy necessitates subject-specific investigation of cerebral blood flow. Occlusion or absence of vessel branches can disrupt communication between the internal carotid and vertebral arteries [4]. Recent numerical studies on intracranial hemodynamics have associated parameters such as wall shear stresses (WSS) or oscillatory shear with pathological effects [5, 6]. However, models within these studies usually cover only one inlet and a limited number of outlets in the proximity of the IA. Furthermore, neuroradiologists rarely rely on image-based blood flow simulations due to the lack of patient-specific flow conditions and unrealistic geometry information [7], although numerous studies have investigated the influence of the inlet and outlet flow conditions on the intracranial hemodynamics [8–11].

Despite being acknowledged as a critical aspect for achieving realistic simulations [12, 13], previous studies on patient-specific hemodynamics have been constrained by limitations imposed on the complexity of the considered vascular geometry. Blood flow simulations for IAs typically include only the parental vessel branch and a short part of the adjacent structures [14]. More complex vessel trees (e.g., the CoW) were investigated, but with restricted regions of interest and a limited number of outlets [13, 15–17]. However, advancements in medical image acquisition allow for higher resolution and accurate segmentation of small vessel branches, enabling consideration of a larger number of outflow cross sections.

**Fig. 1:**
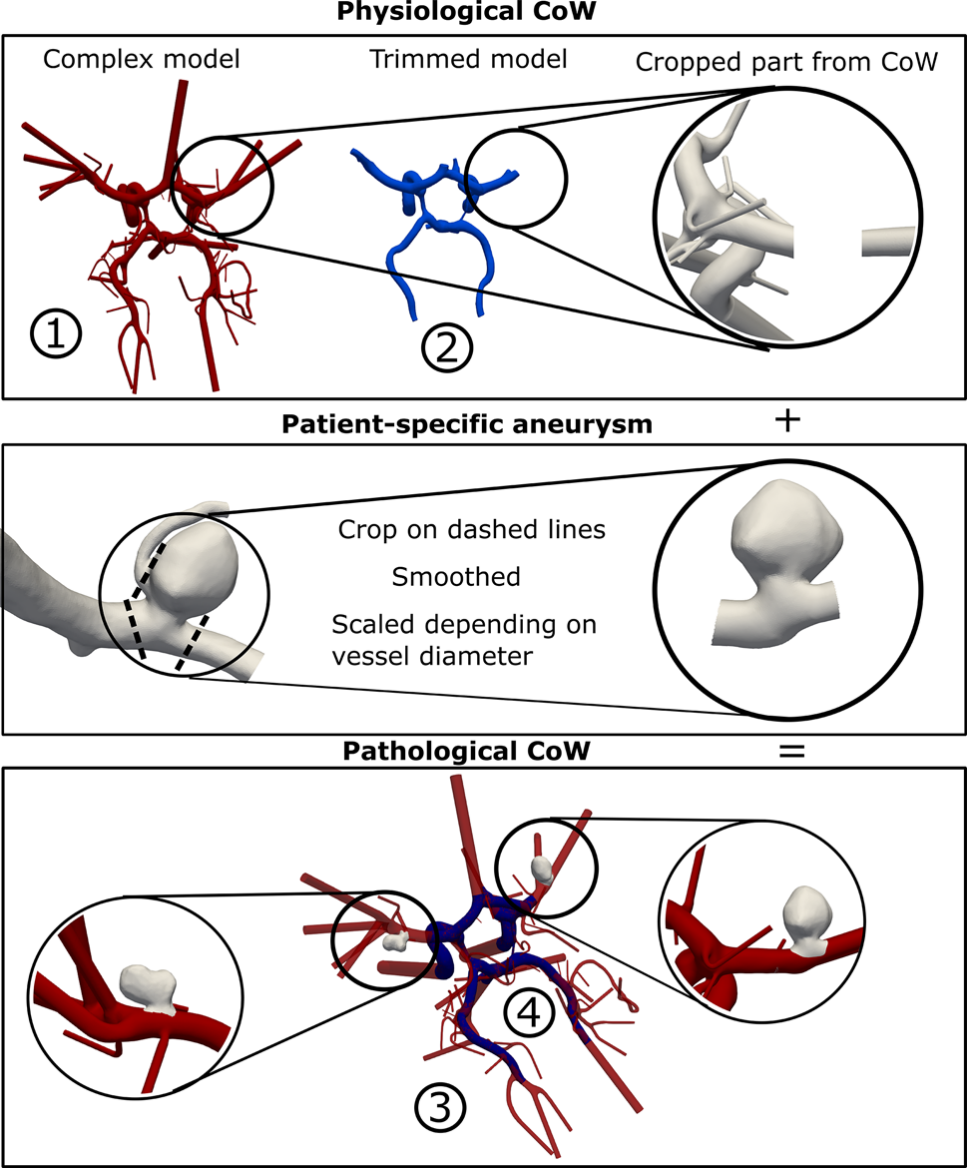
Visualization of the complex (1: red) and trimmed (2: blue) physiological CoW models (top) and the pipeline to create the pathological model: The vessel part was cropped from the CoW, and the IA vessel was placed accordingly. The patient-specific IAs were cropped and scaled to fit the CoW vessel diameter (middle). Afterward, the edited IAs were joined to the CoW model and smoothed to fit. Visualization of the final pathological models (bottom), complex (3: red) and trimmed version (4: blue)

Following a sophisticated segmentation to generate a patient-specific complex CoW geometry (Fig. 1), this study analyzes the effect of the model complexity on the intra-vessel and intra-aneurysmal hemodynamics. High-resolution 7T time-of-flight (ToF) MRI data are used to create a patient-specific 3D surface mesh with 60-outlet vessel cross sections. Blood flow simulations are conducted on this complex geometry model and a trimmed version. Additionally, a pathological variant with two patient-specific intracranial aneurysms is created to examine flow alterations in a clinically relevant scenario. This assessment aims to evaluate the impact of model complexity on the accurate representation of flow- and shear-related parameters in intra-vessel and intra-aneurysmal flow.

## Materials and methods

### Reconstruction of the complex circle of Willis vasculature

The 3D surface segmentation was extracted from high-resolution (0.32 mm isotropic) ToF imaging data from a healthy volunteer. The data were acquired with a 7 Tesla whole-body MRI system (Siemens Healthineers, Erlangen, Germany) including prospective motion correction to prevent image blurring, hence preventing the loss of small vessels [18]. For the 3D models, a multiscale vessel enhancement filtering [19] was applied to the 7T ToF MRI data. Six scales (*σ* = 1…6) were extracted and combined into an intermediate dataset by using the maximum of the six filter responses. Next, seed points were placed manually in the three inlet arteries within the intermediate dataset. All voxels with a maximum of 2% intensity changes (with respect to the seed points) are added to a mask starting from the three seed points. Based on this segmentation mask, marching cubes are applied to extract a 3D surface model. Laplacian smoothing is carried out to smooth the 3D surface model. Afterward, manual post-processing is applied to exclude artifacts or unconnected surface parts. Finally, the inlets and outlets of the 3D vessel model were cut perpendicular to the vessel centerline and extruded to reduce the influence of boundary conditions. The resulting, complex 60-outlet geometry was trimmed to a surface model including only the 10 most prominent outlet cross sections in order to generate a comparison that is equivalent to the current state-of-the-art computational fluid dynamics (CFD) studies in a CoW geometry. Model parameters of the three branches right and left internal carotid artery (ICAr and ICAl) and the basilar artery (BA) are given in Table 1.

**Table 1:** Model parameters including the inlet cross-sectional area, mean volumetric inflow rate, number of outlets and sum of outlet cross-sectional areas of each of the three branches (ICAl, ICAr and BA)

	ICAr	ICAl	BA
Inlet area (mm^2^)	8.76	2.07	2.00
Mean volumetric inflow rate (l/s)	3.434 × 10^–3^	3.065 × 10^–3^	2.332 × 10^–3^

	Trim./Comp	Trim./Comp	Trim./Comp
Number of outlets	4/17	2/9	4/34
Summarized outlet area (mm^2^)	21.76/16.96	8.86/26.85	15.17/27.47

The outlet parameters are divided into trimmed (trim.) and complex (comp.) model and vary among the three inlet branches

### Generation of the pathological model

Two patient-specific IAs were manually incorporated into the healthy CoW model to analyze the impact of model complexity in a clinically relevant scenario. These IAs were selected from a previous international study (MATCH) [20]. The first IA (A) is located at the right middle cerebral artery (MCA) and the second IA (B) at the left MCA. The fusion process involved transforming the IAs to match their exact positions within both CoW models and scaling the IAs to ensure their parent vessel diameter corresponded to the CoW vessel diameter (see Fig. 1).

## Numerical simulations

Four hemodynamic simulations were performed using the finite volume solver STAR CCM + (StarCCM + 2021.3 v16.6, Siemens Product Lifecycle Management Software Inc., Plano, TX, USA) based on the subject-specific geometry models. Blood was assumed to be Newtonian and incompressible, with a dynamic viscosity of 4 mPa·s and a density of 1055 kg/m^3^. Laminar flow and time-resolved simulations with a temporal discretization of 1 ms were conducted over three cycles, with the last cycle considered for evaluation. Spatial discretization was set at a base size of 0.125 mm, resulting in different cell counts for each model (physiological: complex model 4.7 million cells, trimmed model 2.5 million cells; pathological: complex model 6.3 million cells, trimmed model 2.6 million cells).

**Fig. 2:**
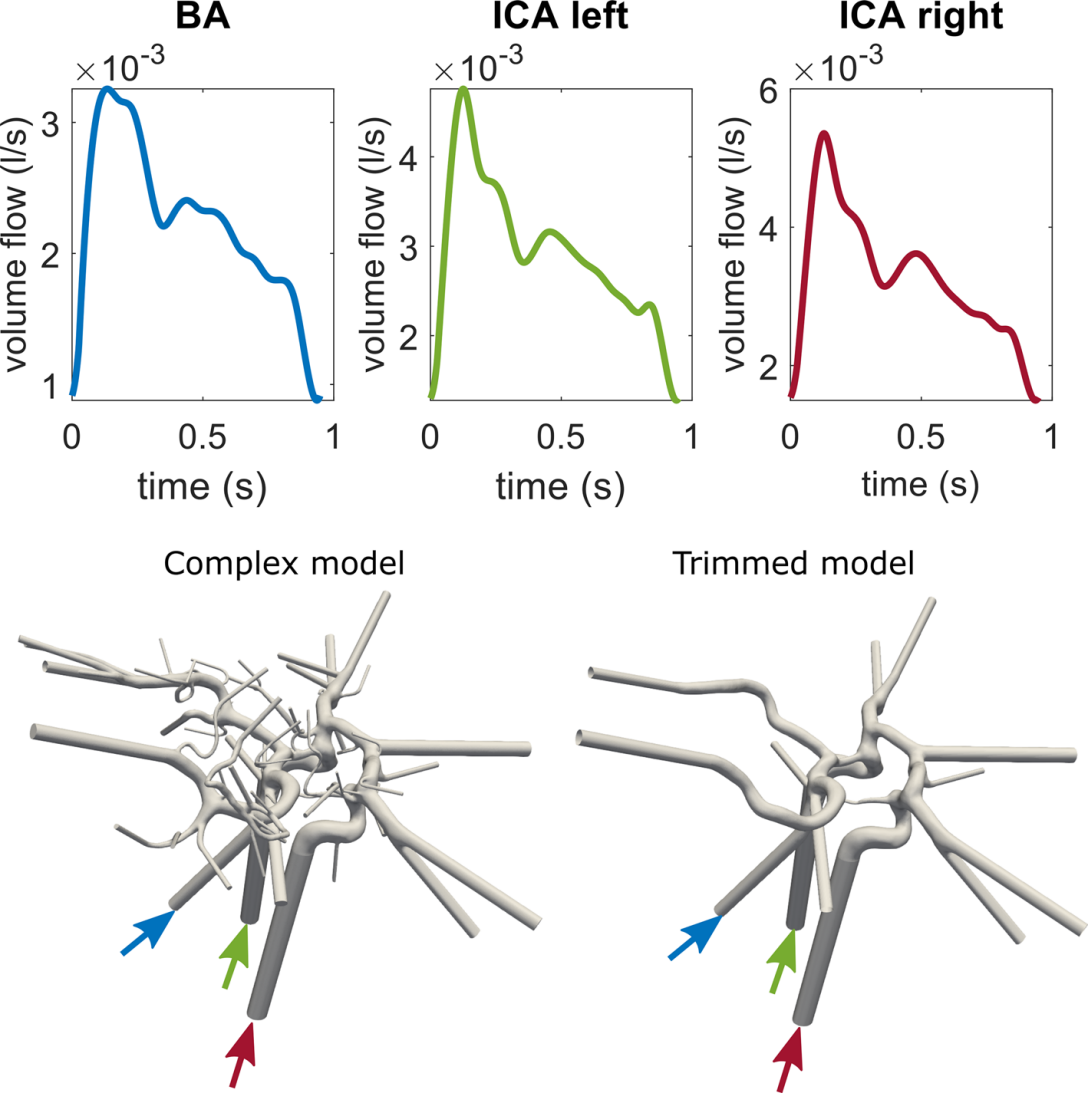
Volume flow rates (l/s) over time (s) used as inlet boundary conditions for all models. The physiological trimmed and complex models are shown below, with color-coded arrows pointing at the corresponding inlets

Each model included three inlets located at specific arteries: basilar artery (BA), right internal carotid artery (ICAr) and left internal carotid artery (ICAl), respectively. Inlet boundary conditions for the simulations were determined using flow rates extracted from volunteer phase-contrast magnetic resonance imaging (PC-MRI) measurements [16, 21]. Figure 2 shows the measured volume flow rates at each inlet (BA: blue, ICAl: green, ICAr: red). As outlet boundary condition, the splitting method based on Murray's law with exponent *n* = 2 was applied [11].

### Data analysis

The evaluation was performed using Ensight (ANSYS Inc., Canonsburg, PA, USA) and MATLAB (MATLAB R2022a, The MathWorks, Natick, USA). Time-averaged velocity values were compared on representative cross sections in the major CoW vessels and their centerline velocities (*CL-v)*. To assess the shape of the flow distribution in the plane-wise evaluation, the velocity distribution was normalized by the corresponding area (*A*) and volumetric flow rate (*Q*).

$$ V_{{{\text{Norm}}}} = \frac{{V \times A_{{{\text{plane}}}} }}{{Q_{{{\text{plane}}}} }} $$

To assess the intra-saccular blood flow within the IAs, wall shear stresses (WSS) at the IA wall and the neck inflow rates (NIR) were evaluated. For a comparative basis, the chosen parameters' time-averaged WSS (TAWSS), oscillatory shear index (OSI) and velocity (*v*) were normalized by their spatial mean within the corresponding IA.

## Results

### Hemodynamics in the complex and trimmed circle of Willis models

**Fig. 3:**
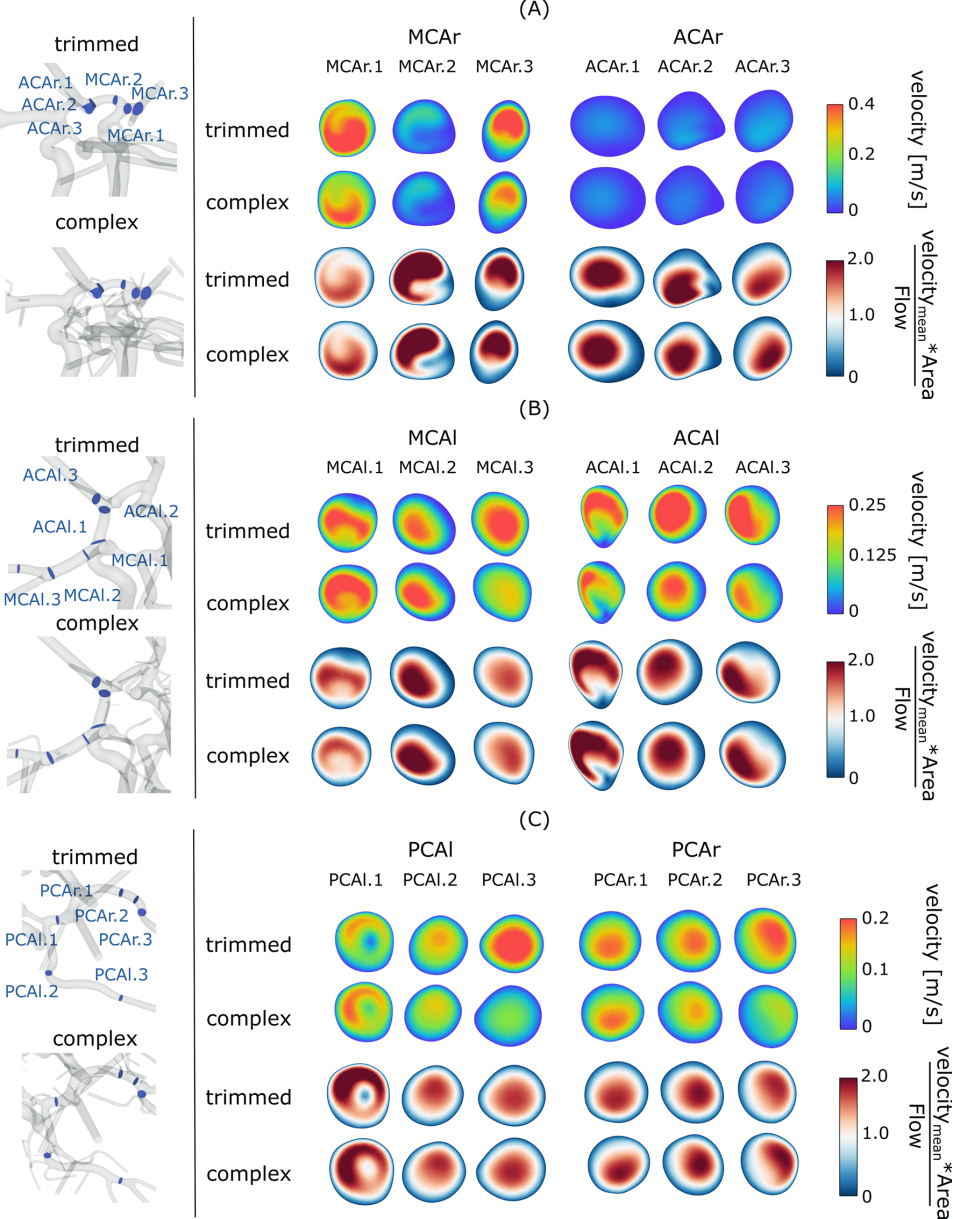
Qualitative visualization of the intra-vessel flow within trimmed and complex model in vessels originating from the right ICA (**a**), the left ICA (**b**) and the BA (**c**). The position of the planes is displayed on the left. On the right, the time-averaged velocity magnitude of three representative is displayed (two top rows). Below, the normalized velocity for the same planes is visualized

Cross sections representing the qualitative velocity distribution in the right ICA show slower values across all planes in the complex models (Fig. 3). Both models show higher velocity magnitudes in the middle cerebral artery (MCA) compared to the anterior cerebral artery (ACA). While differences between the models are noticeable in MCA planes with higher velocities, the lower velocity values in the ACA show fewer absolute differences between the models. The shape of the normalized velocity distribution is similar in the MCA section of the right ICA for both models, but differences are observed in all three ACA planes. In plane ACAr.1, the flow profile is broader, and in plane ACAr.2, a more complex shape of the flow profile is evident in the trimmed model. Additionally, the profile in ACAr.3 is attached to the vessel wall on the right in the complex model but more confined and directed toward the bottom in the trimmed model.

In the left ICA, the qualitative velocity distributions are more consistent (Fig. 3b). However, notable shape differences are observed after normalization. While the right side showed differences in the ACA vessel, the left side exhibits more prominent differences in the MCA. In plane MCAl.1, the complex model has a round flow shape, while the trimmed model shows a more compound "two-peak" shape. Additionally, the flow in planes MCAl.2 and MCAl.3 is more directed toward the right in the complex model. The presence of four additional outlets in the left MCA compared to only one additional outlet in the right MCA results in higher flow profile differences on the left side when comparing complex and trimmed versions. When comparing the left and right side of the CoW geometry, an inconsistent distribution of the flow between MCA and ACA on both sides can be seen. While for the left side of the ACA and MCA the flow is nearly equally distributed (1.2 and 1.3 ml/s for the complex model), the right side shows a larger difference between ACA and MCA flow (0.31 and 1.6 ml/s for the complex model). The result of this uneven distribution is a higher MCA flow on the right side of the CoW when compared with the left counterpart, which leads to an asymmetric flow distribution. Time-dependent volumetric flow rates and mean flow rates through the planes are given in Supplementary Material S1.

Left and right PCA show qualitatively fever differences not only in the absolute velocity values, but also in the shape of their normalized distributions (Fig. 3c). However, with increasing distance from the BA inlet, the differences increase. The highest difference in the normalized flow rates occurs in plane PCAr.1, with a centered flow in the trimmed model, and a flow attached to the bottom in the complex model.

**Fig. 4:**
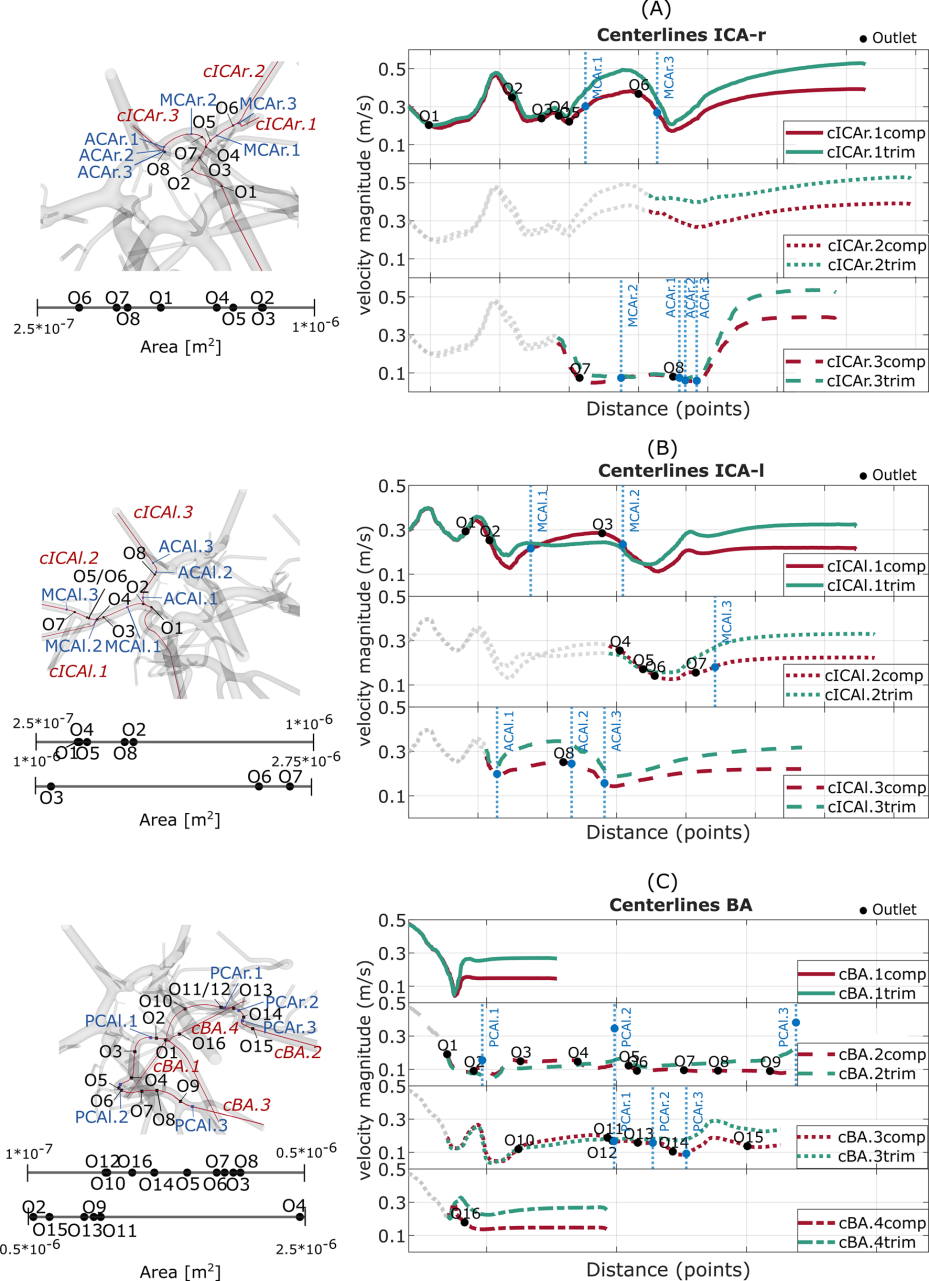
Time-averaged centerline velocities in the right ICA (**a**), the left ICA (**b**) and the BA (**c**). The black dots represent positions at which outlets occur in the complex model (sorted by area on the left). The blue dots visualize the positions of the representative planes in Fig. 3. The velocities are plotted over equidistant centerline points with increasing distance from the inlets. Centerlines (*CL-vs*) are indicated by the letter c and annotated in red or green, while planes are displayed in blue. Outlet, plane and centerline positions are only shown for the complex model, but placed equally in the trimmed version

For the right ICA (Fig. 4a), the *CL-v*s remain higher for the trimmed model over the whole distance. In the ICAr.1, the first four outlets only lead to slight changes of the centerline velocity in the complex model. However, after the fifth outlet, located in the bifurcation separating the ACA and the MCA part of the right ICA, the centerlines of ICAr.1 and ICAr.2 strongly diverge, up to a difference of over 0.1 m/s. The centerline ICAr.3 covers the right ACA vessel. Although the additional outlets (O7 and O8) are smaller than most of the outlets in the MCA branch, the *CL-vs* distal to outlet 8 start to diverge up to a difference of 0.15 m/s. Outlet 8 is located right proximal to the anterior communicating artery (Acom), that connects the left and right side of the CoW. While observing mostly higher values in the trimmed centerlines, consistent with the findings in Fig. 3, the left ICA shows higher velocities for the complex centerline between planes MCAl.1 and MCAl.2. At the centerline ICAl.2 in the MCA portion, the larger outlets (O6 and O7) have a higher influence on the flow changes than the smaller outlets (O4 and O5). In the centerline ICAl.3, the highest difference (0.1 m/s) between both models occurs after plane ACAl.1, located distal to the bifurcation separating the left MCA and ACA. The *CL-vs* of the BA (Fig. 4) are more consistent than the ones in the ICAs. However, centerline BA.2 and BA.3 have vessel segments (after O3 and O10) with the complex *CL-vs* being higher than the trimmed values. Furthermore, with increasing distance from the inlet, the BA centerlines start to diverge more, consistent with the findings in Fig. 3. Additionally, the larger outlets (O13, O9, O11 and O4) have a stronger influence on the difference in the *CL-vs*.

### Hemodynamics within the subject-specific IAs of the pathological circle of Willis model

**Fig. 5:**
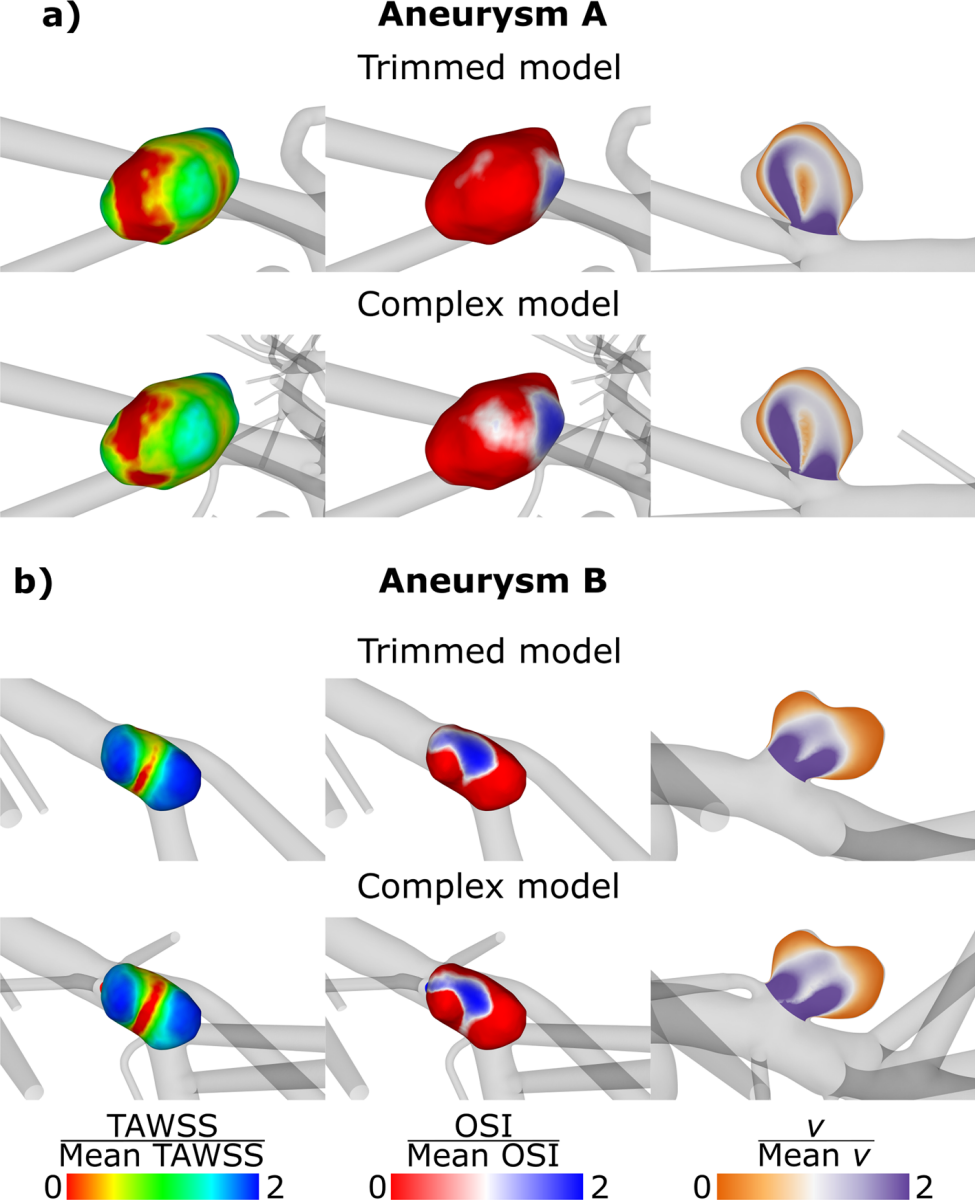
Qualitative visualization of hemodynamics within the IAs A (**a**) and B (**b**). TAWSS normalized by the spatial mean TAWSS inside the according IA, OSI normalized by the spatial mean OSI within the according IA, velocity plane through IA colored by v normalized by spatial mean v within the according IA

Qualitatively, the comparison of the pathological versions features differences particularly on the IAs surfaces (Fig. 5). Within the complex model, a lower TAWSS compared to the trimmed model occurs in IA-A, but a higher TAWSS occurs in IA-B. OSI decreases for both IAs within the complex model compared to the trimmed one. Looking at the flow field within the velocity planes inside the IAs, no essential differences can be noticed (Fig. 5, right column). Still, the temporal mean velocity *v* within the complex model is slightly lower.

**Fig. 6:**
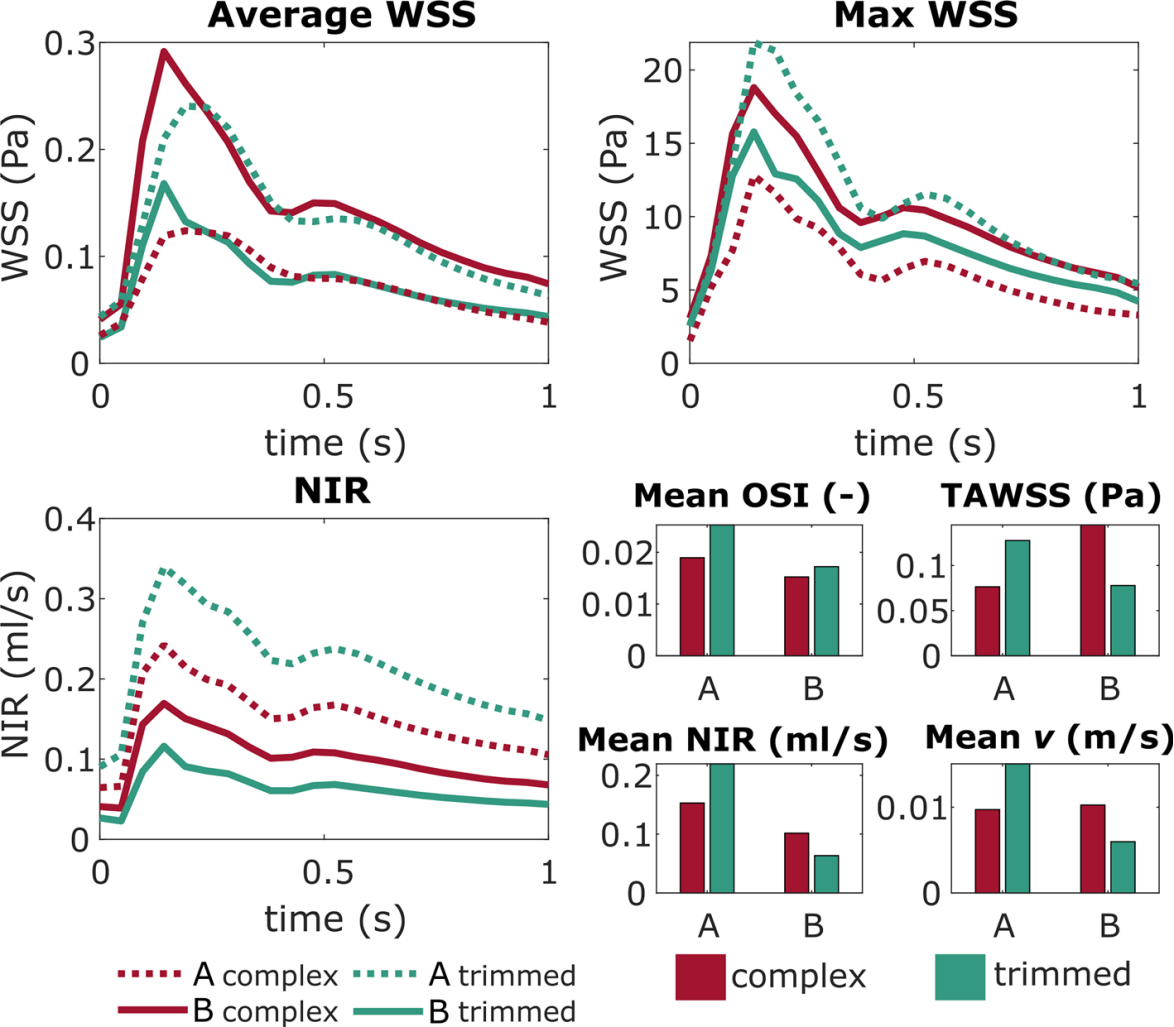
WSS plots over time for spatial mean (average), maximum (max) and NIR inside IA-A (dashed lines) and B (solid lines) comparative for the complex (red) and trimmed (green) model. Spatially and temporally averaged resulting OSI, TAWSS, NIR and v in bar plots (bottom right)

The average and maximum WSS as well as the NIR curves are shown in Fig. 6 within the complex and trimmed model. Average WSS curves feature an equal distribution for each IA. Nevertheless, after model reduction the average WSS increases for IA-A and decreases for IA-B. This is also the case for the maximum WSS of IA-A. For IA-B, the maximum WSS decreases slightly less, particularly during systole. The resulting NIR are not crucially different in course, however present a similar tendency as the average and maximum WSS: NIR into IA-A increases and into IA-B decreases after model trimming. Mean values of TAWSS, NIR and *v* represent the findings that, with model trimming, the parameter values increase for IA-A and decrease for IA-B. Only the OSI values increase for both IAs after model trimming, as also presented qualitatively (Fig. 5).

## Discussion

Flow- and shear-related hemodynamics play a significant role in the formation and growth of intracranial aneurysms (IAs). However, the acceptance of recent image-based blood flow simulations for assessing intra-aneurysmal flow is limited due to the presence of partly unrealistic boundary conditions. To our knowledge, this is the first study focusing on the impact of a highly complex CoW model in the intracranial hemodynamics and featuring a detailed comparison between an extensively segmented, subject-specific model with 60 outlets and a trimmed model version. By manually adding patient-based IAs, the resulting differences in intra-aneurysmal hemodynamics could be investigated directly, visualizing possible clinical relevant flow changes. The highly complex CoW model is an advancement on previous studies that focused only on the major vessel branches [4, 15, 22].

Intra-vessel flow analysis revealed lower velocities due to the outlet-related flow reduction in the complex model. However, certain vessel parts, such as MCAl.1, MCAl.2 and PCAl.1, showed higher velocities. These parts are located near bifurcations (e.g., the bifurcation separating left MCA and ACA) separating major CoW vessels. The *CL-vs* in the ICAl.1 (MCA branch) and ICAl.3 (ACA branch) at the described bifurcation indicate that the flow increases in the MCA branch to the same extent as it decreases in the ACA branch. Thus, depending on the outlet splitting algorithm used, the reduction or inclusion of different outlets can lead to a change in flow distribution between the major CoW vessel branches. These results are reflected in the analysis of the two patient-specific IAs. For IA-A, located at the right MCA, the flow is lower within the complex model. IA-B is located at the left MCA and reveals higher hemodynamics (AWSS, maximum WSS, NIR, *v*) within the complex model, only the OSI decreases here. However, OSI is not necessarily affected by the volumetric flow rate [23].

Nevertheless, the overall lower volumetric flow rates through vessels located on the right and further back left arteries leads to the assumption of a general overestimation of the flow in a trimmed model. This overestimation is usually more present with increasing distance from the inlet vessels as the number of outlets neglected also increases (see Fig. 4c). Differences in model complexity lead to differences in flow magnitude, which is particularly relevant for evaluating pathologies in the distal part of the CoW, such as IAs. These findings can be related to Castro et al. [24], who investigated the impact on hemodynamics when parent arteries are truncated. In previous studies correlating IA geometry and hemodynamics to IA rupture, the model complexity was not taken into account [25, 26]. Thus, specific findings suggesting that the formation and rupture of IAs are solely caused by specific hemodynamic thresholds could have led to misleading conclusions.

Furthermore, independent from the absolute flow values, differences in the flow distribution were noted. The shape of the normalized velocity distribution (Fig. 3) strongly depends on the location analyzed, since higher differences appear close to bifurcations and outlets—additionally shown by the centerline graphs. With higher outlet diameters, the possible influence onto the flow pattern is increased. This is in line with Ren et al. [13] and Alnas et al. [12], who determined that blood flow and WSS is affected by anatomical variations of the CoW. Locations with higher WSS are related to IA occurrence and are thought to carry a higher risk of IA formation. Within this study, the TAWSS distribution in IA-A showed higher variability when more outlets are taken into account, representing a more complex geometry. This is in line with Shen et al. [27] and Valen-Sendstad et al. [14] stating that the intra-aneurysmal flow mainly depends on the parent vessel flow and local geometry.

In the context of analyzing IA hemodynamics and disease progression, it is indispensable to incorporate realistic and patient-specific geometries. The findings of this study emphasize the importance of aneurysm location with respect to the complexity needed in numerical simulations. Moreover, the location of the IA is decisive if a high complex segmentation is crucial. The IAs analyzed in this study are located at the right and left MCA and show influence from the model complexity. As IAs statistically occur mostly at the anterior circulation (ICA junction to PCA, MCA trifurcation, ACA complex) [28], this should be taken into consideration when studying the risks of IA formation and rupture, particularly when correlating these risks with hemodynamics. Previous studies have demonstrated varying results in relation to this aspect [29, 30]. It is important to note that the assessment of intra-aneurysmal hemodynamics has clinical relevance with respect to treatment planning, risk assessment and outcome prediction.

This study has several limitations. First, the inlet boundary condition derived from PC-MRI measurements features low resolution and therefore may result in an underestimation. Nevertheless, the waveform can be assumed to be as subject-specific as possible due to the data acquisition occurring during MRI scans. Second, only one case is taken into account within this study. However, this case provides a complex structure which has not been published in the literature before, and the segmentation process required considerable time and effort. Finally, although the IAs featured are certainly patient-specific, they are added onto a healthy CoW model. Moreover, the inflow rates are waveforms from the same healthy subject. Nevertheless, the addition of the IAs was performed by ensuring the preservation of the IA neck and by scaling the IA to the equivalent parent vessel diameter.

As can be derived from the results, the model complexity influences the flow magnitudes and the flow distribution. A complex model is advantageous and should be used in image-based blood flow simulations of IAs if possible, especially in the vicinity of and in proximity to the IA. Specifically, local differences in complexity mainly influences the shape of the flow distribution. However, global complexity influences the magnitude of velocities in the model considered.

Future work will include a larger number of cases to strengthen the findings of this study. Furthermore, this can be accomplished by adding IAs in different locations to discover the impact of the altered flow on the intra-aneurysmal hemodynamics within the ICA or ACA, for instance. Accessing the imaging data of a patient-specific pathological CoW would provide the opportunity to analyze IA hemodynamics in a realistic and not artificially created pathological model, which would ensure the pathological inflow conditions as well.

## Conclusion

In this study, the influence of geometric complexity on intra-vessel and intra-aneurysmal flow was shown and, for the first time, analyzed in detail in a 60-outlet CoW model. Both flow magnitude and the shape of the flow distribution depend on the number of outlets in the model. The first is mainly influenced by the global vessel model, whereas the latter depends on the local structure. Moreover, the location of IAs is critical with respect to the sensitivity of flow- and shear-related parameters in the aneurysm to complexity.

### Supplementary Information

Below is the link to the electronic supplementary material.

Supplementary file1 (DOCX 454 KB)

## Data Availability

Processed data and code are available upon reasonable request from the corresponding author.
