# Supplementary material for: Image-based hemodynamic simulations for intracranial aneurysms: the impact of complex vasculature
Source: Int J Comput Assist Radiol Surg. 2024 Jan 11;19(4):687–97. doi: 10.1007/s11548-023-03045-3 (PMC10973067; doi:10.1007/s11548-023-03045-3)
Supplement: Supplementary file 1 — Supplementary file1 (DOCX 454 KB) [file 11548_2023_3045_MOESM1_ESM.docx]

# Supplementary

## S1. Time-averaged flow rates

The time-dependent volumetric flow rates through the planes (Fig. 3) are displayed in Figure S1. Mean flow values are calculated in Table S1.


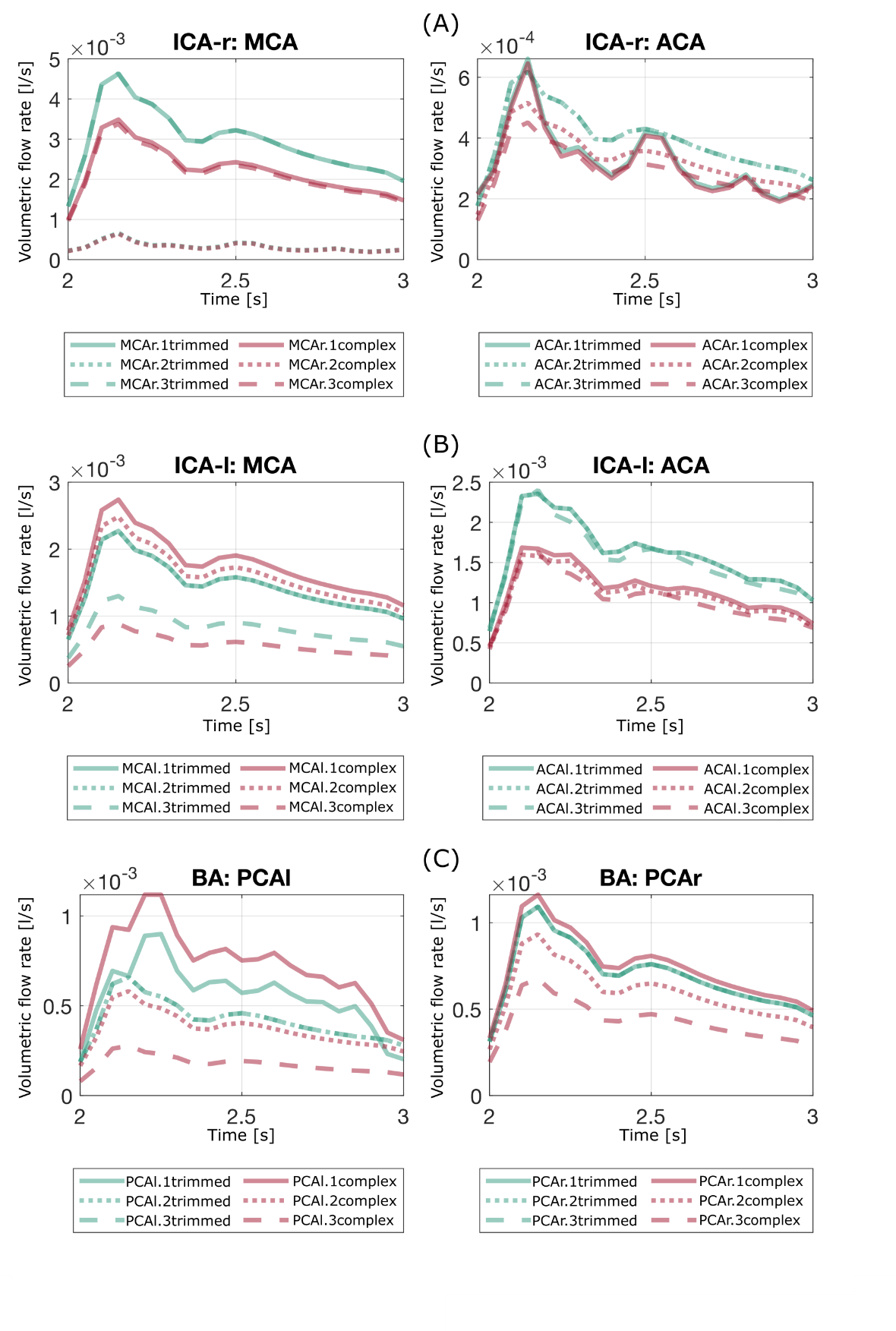


Figure S1: Time-dependent flow rates through representative planes for the complex and trimmed CoW model.

Table S1: Temporal mean flow rates in the main vessel branches.

| **Vessel branch** | **Mean flow (trimmed) [ml/s]** | **Mean flow (complex) [ml/s]** |
| --- | --- | --- |
| MCAr | 2.1 | 1.6 |
| ACAr | 0.37 | 0.31 |
| MCAl | 1.2 | 1.3 |
| ACAl | 1.6 | 1.2 |
| PCAr | 0.69 | 0.59 |
| PCAl | 0.46 | 0.42 |
